# Supplementary material for: Seizures, behavioral deficits, and adverse drug responses in two new genetic mouse models of HCN1 epileptic encephalopathy
Source: eLife. 2022 Aug 16;11:e70826. doi: 10.7554/eLife.70826 (PMC9481245; doi:10.7554/eLife.70826)
Supplement: Figure 2—source data 1. — Number of animals is indicated in parentheses. Times in border and center zones, respectively, are expressed as percentage of total trial duration. *Data was analyzed with a Mann–Whitney U test; #data was analyzed with a Student’s t-test. Data represent mean ± SEM. [file elife-70826-fig2-data1.docx]

| **Parameter** | **WT (n = 29)** | ***Hcn1^GD/+^* (n = 22)** | ***P* value** |
| --- | --- | --- | --- |
| Running speed (cm/s) | 6.85 ± 0.42 | 10.72 ± 1.34 | 0.0001* |
| Distance moved (m) | 60.90 ± 3.78 | 95.12 ± 12.02 | 0.0003* |
| Occupation ratio | –0.77 ± 0.02 | –0.89 ± 0.02 | 0.0002* |
| Latency to center (s) | 23.70 ± 4.89 | 47.03 ± 16.29 | 0.568* |
| Time in border (%) | 55.29 ± 2.16 | 74.98 ± 3.47 | < 0.001* |
| Time in center (%) | 6.65 ± 0.50 | 3.65 ± 0.66 | 0.0016* |
| Turns (n/min) | 0.95 ± 0.07 | 2.18 ± 0.56 | 0.003* |
| **Parameter** | **WT (n = 22)** | ***Hcn1^MI/+^* (n = 21)** | ***P* value** |
| Running speed (cm/s) | 8.69 ± 0.49 | 11.61 ± 0.69 | 0.0013^#^ |
| Distance moved (m) | 68.56 ± 3.43 | 100.00 ± 5.05 | < 0.001^#^ |
| Occupation ratio | –7.28 ± 0.04 | –0.89 ± 0.01 | 0.0002* |
| Latency to center (s) | 24.87 ± 6.26 | 36.14 ± 7.71 | 0.262* |
| Time in border (%) | 54.44 ± 2.29 | 67.67 ± 1.90 | < 0.001^#^ |
| Time in center (%) | 8.17 ± 1.07 | 3.93 ± 0.43 | 0.0016* |
| Turns (n/min) | 0.95 ± 0.08 | 1.49 ± 0.16 | 0.0018* |
