# Supplementary material for: Seizures, behavioral deficits, and adverse drug responses in two new genetic mouse models of HCN1 epileptic encephalopathy
Source: eLife. 2022 Aug 16;11:e70826. doi: 10.7554/eLife.70826 (PMC9481245; doi:10.7554/eLife.70826)
Supplement: Figure 2—source data 2. — Parameters listed correspond to the right front paw. Number of animals is shown in parentheses. *Data was analyzed with a Mann–Whitney U test; #data was analyzed with a Student’s t-test. Data represent mean ± SEM. [file elife-70826-fig2-data2.docx]

| **Parameter** | **Speed range** | **WT** | ***Hcn1^GD/+^*** | ***P* value** |
| --- | --- | --- | --- | --- |
| Running speed (cm/s) | all | 21.40 ± 0.91 (n = 30) | 34.48 ± 1.59 (n = 21) | < 0.001^#^ |
| BOS front (cm) | 20-30 cm/s | 1.17 ± 0.03 (n = 22) | 1.37 ± 0.06 (n = 9) | 0.005^#^ |
| BOS hind (cm) | 20-30 cm/s | 2.05 ± 0.05 (n = 22) | 1.95 ± 0.04 (n = 9) | 0.272^#^ |
| Stride length (cm) | 20-30 cm/s | 5.97 ± 0.18 (n = 22) | 5.90 ± 0.19 (n = 9) | 0.685* |
| Max. contact area (cm^2^) | 20-30 cm/s | 0.19 ± 0.02 (n = 22) | 0.22 ± 0.03 (n = 9) | 0.414* |
| Stand (s) | 20-30 cm/s | 0.11 ± 0.004 (n = 22) | 0.13 ± 0.003 (n = 9) | 0.160* |
| Step cycle (s) | 20-30 cm/s | 0.23 ± 0.007 (n = 22) | 0.23 ± 0.004 (n = 9) | 0.535* |
| Regularity index (%) | 20-30 cm/s | 97.33 ± 1.33 (n = 22) | 96.33 ± 2.37 (n = 9) | 0.786* |
| BOS front (cm) | 30-40 cm/s | 1.15 ± 0.04 (n = 8) | 1.41 ± 0.04 (n = 13) | 0.0004^#^ |
| BOS hind (cm) | 30-40 cm/s | 2.03 ± 0.03 (n = 8) | 1.88 ± 0.03 (n = 13) | 0.006^#^ |
| Stride length (cm) | 30-40 cm/s | 6.90 ± 0.17 (n = 8) | 6.51 ± 0.18 (n = 13) | 0.231* |
| Max. contact area (cm^2^) | 30-40 cm/s | 0.24 ± 0.01 (n = 8) | 0.22 ± 0.03 (n = 13) | 0.546^#^ |
| Stand (s) | 30-40 cm/s | 0.09 ± 0.003 (n = 8) | 0.10 ± 0.004 (n = 13) | 0.210* |
| Step cycle (s) | 30-40 cm/s | 0.19 ± 0.003 (n = 8) | 0.18 ± 0.005 (n = 13) | 0.514^#^ |
| Regularity index (%) | 30-40 cm/s | 96.84 ± 1.45 (n = 8) | 97.73 ± 1.56 (n = 13) | 0.262* |
| **Parameter** | **Speed range** | **WT** | ***Hcn1^MI/+^*** | ***P* value** |
| Running speed (cm/s) | all | 19.99 ± 0.92 (n = 22) | 29.62 ± 1.67 (n = 21) | < 0.001^#^ |
| BOS front (cm) | 20-30 cm/s | 1.14 ± 0.03 (n = 20) | 1.08 ± 0.04 (n = 15) | 0.240^#^ |
| BOS hind (cm) | 20-30 cm/s | 2.02 ± 0.05 (n = 20) | 1.88 ± 0.05 (n = 15) | 0.076^#^ |
| Stride length (cm) | 20-30 cm/s | 6.12 ± 0.09 (n = 20) | 6.47 ± 0.14 (n = 15) | 0.085^#^ |
| Max. contact area (cm^2^) | 20-30 cm/s | 0.20 ± 0.02 (n = 20) | 0.20 ± 0.02 (n = 15) | 0.856* |
| Stand (s) | 20-30 cm/s | 0.12 ± 0.003 (n = 20) | 0.13 ± 0.004 (n = 15) | 0.347* |
| Step cycle (s) | 20-30 cm/s | 0.24 ± 0.004 (n = 20) | 0.24 ± 0.007 (n = 15) | 0.410^#^ |
| Regularity index (%) | 20-30 cm/s | 96.40 ± 0.97 (n = 20) | 96.63 ± 1.40(n = 15) | 0.674* |
| BOS front (cm) | 30-40 cm/s | 1.17 ± 0.03 (n = 6) | 1.14 ± 0.04 (n = 12) | 0.719^#^ |
| BOS hind (cm) | 30-40 cm/s | 1.92 ± 0.14 (n = 6) | 1.73 ± 0.09 (n = 12) | 0.246^#^ |
| Stride length (cm) | 30-40 cm/s | 6.86 ± 0.17 (n = 12) | 6.93 ± 0.15 (n = 6) | 0.782^#^ |
| Max. contact area (cm^2^) | 30-40 cm/s | 0.23 ± 0.04 (n = 12) | 0.22 ± 0.02 (n = 6) | 0.783^#^ |
| Stand (s) | 30-40 cm/s | 0.10 ± 0.006 (n = 12) | 0.09 ± 0.001 (n = 6) | 0.084^#^ |
| Step cycle (s) | 30-40 cm/s | 0.19 ± 0.005 (n = 12) | 0.19 ± 0.004 (n = 6) | 0.148^#^ |
| Regularity index (%) | 30-40 cm/s | 97.78 ± 1.81 (n = 12) | 99.54 ± 0.31(n = 6) | 0.461* |
