# Supplementary material for: Seizures, behavioral deficits, and adverse drug responses in two new genetic mouse models of HCN1 epileptic encephalopathy
Source: eLife. 2022 Aug 16;11:e70826. doi: 10.7554/eLife.70826 (PMC9481245; doi:10.7554/eLife.70826)
Supplement: Figure 2—source data 3. — Number of animals is indicated in parentheses. Latency data represent mean ± SEM. [file elife-70826-fig2-data3.docx]

| **Parameter** | **WT (22)** | ***Hcn1^GD/+^*** **(18)** | **WT (11)** | ***Hcn1^MI/+^* (11)** |
| --- | --- | --- | --- | --- |
| Success rate Trial 1 | 20/22 | 1/18 | 11/11 | 11/11 |
| Success rate Trial 2 | 22/22 | 1/18 | 11/11 | 11/11 |
| Success rate Trial 3 | 22/22 | 2/18 | 11/11 | 11/11 |
| Latency Trial 1 (s) | 13.18 ± 0.794 | 7.0 ± 0 | 18.18 ± 2.456 | 20.55 ± 6.768 |
| Latency Trial 2 (s) | 11.05 ± 0.749 | 7.0 ± 0 | 12.64 ± 0.789 | 9.73 ± 0.821 |
| Latency Trial 3 (s) | 10.23 ± 0.431 | 14.5 ± 5.5 | 15.18 ± 2.071 | 14.91 ± 5.888 |
