# Supplementary material for: Seizures, behavioral deficits, and adverse drug responses in two new genetic mouse models of HCN1 epileptic encephalopathy
Source: eLife. 2022 Aug 16;11:e70826. doi: 10.7554/eLife.70826 (PMC9481245; doi:10.7554/eLife.70826)
Supplement: Figure 3—source data 1. — Alternation rates and time to complete 24 transitions are expressed as the mean of 2 days. Number of animals is indicated in parentheses. *Data was analyzed using a Mann–Whitney U test. Data represent mean ± SEM. [file elife-70826-fig3-data1.docx]

| **Parameter** | **WT (22)** | ***Hcn1^GD/+^*** **(18)** | ***P* value** |
| --- | --- | --- | --- |
| Alternation rate (%) | 65.51 ± 1.298 | 46.38 ± 4.069 | < 0.001* |
| Time (min) | 8.11 ± 0.436 | 3.67 ± 0.3201 | < 0.001* |
| **Parameter** | **WT (11)** | ***Hcn1^MI/+^* (11)** | ***P* value** |
| Alternation rate (%) | 63.04 ± 1.909 | 61.01 ± 2.553 | 0.578* |
| Time (min) | 5.72 ± 0.3113 | 3.80 ± 0.2363 | < 0.001* |
