# Supplementary material for: Seizures, behavioral deficits, and adverse drug responses in two new genetic mouse models of HCN1 epileptic encephalopathy
Source: eLife. 2022 Aug 16;11:e70826. doi: 10.7554/eLife.70826 (PMC9481245; doi:10.7554/eLife.70826)
Supplement: Figure 3—source data 2. — Number of animals is indicated in parentheses. *Data was analyzed using a Mann–Whitney U test. Data represent mean ± SEM. [file elife-70826-fig3-data2.docx]

| **Parameter** | **WT (22)** | ***Hcn1^GD/+^*** **(18)** | ***P* value** |
| --- | --- | --- | --- |
| Exploration time familiar object (sec) | 6.769 ± 0.468 | 4.591 ± 0.823 | – |
| Exploration time novel object (sec) | 13.44 ± 0.466 | 15.65 ± 0.831 | – |
| Discrimination index (D.I.) | 0.3303 ± 0.0462 | 0.546 ± 0.0815 | 0.020* |
| **Parameter** | **WT (12)** | ***Hcn1^MI/+^* (16)** | ***P* value** |
| Exploration time familiar object (sec) | 7.020 ± 0.710 | 7.810 ± 0.703 | – |
| Exploration time novel object (sec) | 13.24 ± 0.693 | 12.46 ± 0.701 | – |
| Discrimination index (D.I.) | 0.3078 ± 0.0697 | 0.2293 ± 0.0693 | 0.732* |
