# Supplementary material for: Seizures, behavioral deficits, and adverse drug responses in two new genetic mouse models of HCN1 epileptic encephalopathy
Source: eLife. 2022 Aug 16;11:e70826. doi: 10.7554/eLife.70826 (PMC9481245; doi:10.7554/eLife.70826)
Supplement: Figure 5—source data 1. — Number of cells is shown in parenthesis. Number of animals used for GD: WT n = 6 mice, GD n = 5 mice; and for MI: WT n = 3 mice, MI n = 3 mice. *Data was analyzed using a Mann–Whitney U test. Data represent mean ± SEM. [file elife-70826-fig5-data1.docx]

| **Parameter** | **WT (19)** | ***Hcn1^GD/+^*** **(21)** | ***P* value** |
| --- | --- | --- | --- |
| AP Threshold (mV) | –44.52 ± 0.908 | –47.08 ± 1.009 | 0.106* |
| AP Peak (mV) | 47.01 ± 1.246 | 46.28 ± 1.483 | 0.606* |
| AP Width (ms) | 1.462 ± 0.044 | 1.493 ± 0.032 | 0.634* |
| **Parameter** | **WT (11)** | ***Hcn1^MI/+^* (12)** | ***P* value** |
| AP Threshold (mV) | –49.12 ± 0.866 | –48.03 ± 1.191 | 0.496* |
| AP Peak (mV) | 48.85 ± 1.907 | 52.63 ± 1.098 | 0.114* |
| AP Width (ms) | 1.415 ± 0.059 | 1.388 ± 0.032 | 0.684* |
