# Supplementary material for: Seizures, behavioral deficits, and adverse drug responses in two new genetic mouse models of HCN1 epileptic encephalopathy
Source: eLife. 2022 Aug 16;11:e70826. doi: 10.7554/eLife.70826 (PMC9481245; doi:10.7554/eLife.70826)
Supplement: Figure 9—source data 1. — Number of cells is indicated in parenthesis. #Data was analyzed with a Student’s t-test. Data represent mean ± SEM. [file elife-70826-fig9-data1.docx]

| **V_1/2_ (mV)** | **– Lamotrigine** | ***+* Lamotrigine** | ***P* value** |
| --- | --- | --- | --- |
| HCN1 | –71.7 ± 1.5 (n = 4) | –72.6 ± 1 (n = 4) | 0.635^#^ |
| HCN1 + TRIP8b | –81.7 ± 1.4 (n = 5) | –80.8 ± 1.1 (n = 5) | 0.928^#^ |
| HCN2 | –97.6 ± 1.4 (n = 5) | –99.4 ± 1.1 (n = 5) | 0.342^#^ |
| **Tail current density (pA/pF)** | **– Lamotrigine** | ***+* Lamotrigine** | ***P* value** |
| HCN1 | 8.3 ± 1.95 (n = 4) | 9 ± 2.1 (n = 4) | 0.815^#^ |
| HCN1 + TRIP8b | 18.2 ± 1.3 (n = 5) | 18.7 ± 1.7 (n = 5) | 0.821^#^ |
| HCN2 | 18.2 ± 2.1 (n = 5) | 16 ± 1.65 (n = 5) | 0.434^#^ |
