# Supplementary material for: Seizures, behavioral deficits, and adverse drug responses in two new genetic mouse models of HCN1 epileptic encephalopathy
Source: eLife. 2022 Aug 16;11:e70826. doi: 10.7554/eLife.70826 (PMC9481245; doi:10.7554/eLife.70826)
Supplement: Figure 10—source data 1. — Number of cells is shown in parenthesis. *Data was analyzed with a paired t-test. #Data was analyzed with a Wilcoxon matched-pairs signed-rank test. Data represent mean ± SEM. [file elife-70826-fig10-data1.docx]

| **Parameter** | **-ZD** | **+ZD** | ***P* value** |
| --- | --- | --- | --- |
| Sag ratio WT | 0.78 ± 0.02 (n = 10) | 0.96 ± 0.02 (n = 8) | < 0.001* |
| Sag ratio *Hcn^GD/+^* | 0.96 ± 0.01 (n = 8) | 0.98 ± 0.01 (n = 6) | 0.188^#^ |
| RMP (mV) WT | –69.36 ± 0.83 (n = 10) | –72.67 ± 0.88 (n = 8) | 0.010* |
| RMP (mV) *Hcn^GD/+^* | –63.85 ± 1.14 (n = 8) | –66.53 ± 0.99 (n = 6) | 0.156^#^ |
| R_in_ (MOhm) WT | 148.50 ± 11.79 (n = 10) | 238.90 ± 26.89 (n = 8) | 0.004* |
| R_in_ (MOhm) *Hcn^GD/+^* | 147.00 ± 9.79 (n = 8) | 144.20 ± 8.91 (n = 6) | 0.688^#^ |
