# Supplementary material for: Seizures, behavioral deficits, and adverse drug responses in two new genetic mouse models of HCN1 epileptic encephalopathy
Source: eLife. 2022 Aug 16;11:e70826. doi: 10.7554/eLife.70826 (PMC9481245; doi:10.7554/eLife.70826)
Supplement: Source code 1. — Source code used to plot typical examples of seizures, including the electrocorticogram (ECoG) trace and corresponding time–frequency spectrogram, using the custom written function plot_telemSz_andrea. [file elife-70826-code1.docx]

% Plot typical seizures for paper using the function “plot_telemSz_andrea(fullpath,seg,[],clims,[],ylims)”

% FIGURE 4 A: seizure from Hcn1^GD/+^ animal

fullpath = '/mnt/raid/data_dirk/telemetry/HCN1/analysis/processed/HCNGD_364_2020-05-25_24h'; % the directory of the eeg file

seg = [8720 9040; 100 200];

% add the time of seizure beginning to end in seconds (start = beginning of recording); add the time for zooming into the seizure (start = beginning of seizure)

clims.mtspec = [-68 -20]; %multitaper color clims

clims.cwt = [0.005 0.15]; %wavelet color clims

ylims = [-1.2 1.2];

plot_telemSz_andrea(fullpath,seg,[],clims,[],ylims)

% FIGURE 4 C: plot seizure from Hcn1^MI/+^ animal

fullpath = '/mnt/raid/data_dirk/telemetry/HCN1/analysis/processed/HCNMI_216_2020-05-25_24h';

% the directory of the eeg file

seg = [27280 27530; 100 152];

% add the time of seizure beginning to end in seconds (start = beginning of recording); add the time for zooming into the seizure (start = beginning of seizure)

clims.mtspec = [-68 -20]; %multitaper color clims

clims.cwt = [0.005 0.15]; %wavelet color clims

ylims = [-1.3 1.7];

plot_telemSz_andrea(fullpath,seg,[],clims,[],ylims)

function [] = plot_telemSz_andrea(fullpath,seg,mtwin,clims,mtparams,ylims)

% function [] = plot_telemSz_andrea(fullpath,seg,mtwin,clims,mtparams)

%

% inputs

% - /base/path/todata (should be a todata.eeg, todata.xml in /base/path)

% - seg = [outer_start outer_end; zoomin_start zoomin_end]

% ATTENTION! zoomin_start is an OFFSET from outer_start

% - mtwin = [win_length win_stepsize] in seconds

% - clims.mtspec, clims.cwt each have CLim edges for plots

% - mtparams = % see 'doc mtspectrumc'

% mtparams.Fs = FROM XML

% pixels we want the figure wide and high

wid = 1000; hgt = 640;

if nargin<6, ylims = []; end

if nargin<5 | isempty(mtparams)

mtparams.tapers = [3 5];

mtparams.trialave = 0;

mtparams.fpass = [0.5 80]; %frequency limits

end

if nargin<4 | isempty(clims)

clims.mtspec = [-68 -28]; %multitaper color clims

clims.cwt = [0.005 0.145]; %continuous wavelet color limits

end

if nargin<3 | isempty(mtwin)

mtwin = [2 .5]; % [moving_window_size moving_window_stepsize]; seconds

end

% for xml_load(): https://web-archive.southampton.ac.uk/www.geodise.org/download

addpath('/path/to/xml_toolbox-2.0'); % add path to xml_toolbox-2.0

% for LoadBinary(): http://fmatoolbox.sourceforge.net

addpath(genpath('/path/to/FMAToolbox/')); % add path to FMAToolbox

% for mtspecgramc(): http://chronux.org

addpath(genpath('/path/to/chronux/spectral_analysis')) % add path to chronux/spectral_analysis

% Check for xml, load

if ~exist([fullpath '.xml'])

error('No XML file! Is fullpath correct? Did you open first in neuroscope?');

end

par = xml_load([fullpath '.xml']);

fs = str2num(par.acquisitionSystem.samplingRate); %if this is wrong, so is your specgram y-axis!

vRange = str2num(par.acquisitionSystem.voltageRange);

amplif = str2num(par.acquisitionSystem.amplification);

mVrescale = 2^16/((vRange*1e3)/amplif);

mtparams.Fs = fs; % important!

% load data

eeg = LoadBinary([fullpath '.eeg'],'frequency',fs)./mVrescale;

% area to plot

start = seg(1,1)*fs; % in samples

finish = seg(1,2)*fs;

szBorders = seg(2,:); % in seconds

% compute chronux multitaper specgram

[S,t,f] = mtspecgramc(eeg(start:finish),mtwin,mtparams);

% plot!

figure('DefaultAxesPosition',[0.06 0.06 0.92 0.93],...

'PaperPositionMode','manual','PaperPosition',[0 0 wid/100 hgt/100],...

'Position',[0 0 wid hgt],'DefaultAxesFontSize',14);

subplot(11,1,1:2)

plot(eeg(start:finish),'k','LineWidth',.2)

set(gca,'TickDir','out','XTick',[])

axis tight, box off

ylabel('mV')

if ~isempty(ylims), ylim(ylims); end

subplot(11,1,3:5)

h = pcolor(t,f,10*log10(abs(S'))); box off; % in dB, drop imaginary (phase)

set(h,'EdgeColor','none'); colormap jet

set(gca,'CLim',clims.mtspec) % color limits

ylim([mtparams.fpass(1)+0.5 mtparams.fpass(2)]); % y limits

ylabel('Hz'), xlabel('seconds')

set(gca,'YTick',2.^(1:6)); % y ticks

set(gca,'TickDir','out','YScale','log','YMinorTick','off'); % log scale

pos = get(gca,'Position');

set(gca,'Position',[pos(1) pos(2)+.06 pos(3) pos(4)-.05]);

% draw zoom in lines

line(repmat(szBorders(1),1,2),mtparams.fpass,'LineStyle','--','Color',[.3 .3 .3],'LineWidth',2);

line(repmat(szBorders(2),1,2),mtparams.fpass,'LineStyle','--','Color',[.3 .3 .3],'LineWidth',2);

% draw zoomed-in trace

subplot(11,1,6:7)

eegSnip = eeg((start+fs*szBorders(1)):(start+fs*szBorders(2)));

plot(eegSnip,'k','LineWidth',.2)

axis tight, box off

set(gca,'TickDir','out','XTick',[]), ylabel('mV')

if ~isempty(ylims), ylim(ylims); end

% plot wavelet, use cwt() and resample to 1/10th resolution, pcolor+log scale

subplot(11,1,8:11)

[wtp f] = cwt(eegSnip,fs,'VoicesPerOctave',48,'TimeBandwidth',90,'FrequencyLimits',...

[mtparams.fpass(1)+0.5 mtparams.fpass(2)]);

downsamp_wtp = resample(abs(wtp)',1,10)';

h = pcolor(linspace(szBorders(1),szBorders(2),length(downsamp_wtp)),f,downsamp_wtp);

axis tight, box off

set(h,'EdgeColor','none');

set(gca,'CLim',clims.cwt)

set(gca,'YTick',2.^(1:6));

set(gca,'TickDir','out','YScale','log','YMinorTick','off')

xlabel('seconds')

ylabel('Hz')
