# Supplementary material for: Seizures, behavioral deficits, and adverse drug responses in two new genetic mouse models of HCN1 epileptic encephalopathy
Source: eLife. 2022 Aug 16;11:e70826. doi: 10.7554/eLife.70826 (PMC9481245; doi:10.7554/eLife.70826)
Supplement: Source data 1. — Average intensity (log2), fold change, p-value, and false discovery rate (FDR)-corrected p-value (considering all 22,206 probe sets) were derived using an Affymetrix-based screening of hippocampal tissue from Hcn1GD/+, Hcn1MI/+, and WT mice (n = 4 for each group) for 112 candidate genes, representing all main families of voltage-gated ion channels (see ‘Materials and methods’). Top 10 hits, ranked by p-value, are shown for each comparison. Values for GFAP and vimentin, two genes with an expected increase in hippocampal expression as a result of reactive gliosis (Figure 4—figure supplement 1, Escartin et al., 2021; Stringer, 1996), are also shown for reference. Note that both markers showed a nearly approximately twofold increase in Hcn1GD/+ animals (with FDR-corrected p-value<0.05 in the case of vimentin), while negligible changes were observed in Hcn1MI/+ animals. [file elife-70826-data1.docx]

| **Gene Symbol** | **Description** | **WT** | ***Hcn1^GD/+^*** | **Fold change** | ***P* value** | **FDR *P* value** |
| --- | --- | --- | --- | --- | --- | --- |
| **Voltage-gated ion channels** | | | | | | |
| Kcnq3 | K_V_7.3 | 13.14 | 12.57 | –1.48 | 0.0007 | 0.2286 |
| Kcnh1 | K_V_10.1/Eag1 | 10.25 | 10.82 | 1.49 | 0.0023 | 0.2905 |
| Trpa1 | TRPA1 | 4.33 | 3.77 | –1.47 | 0.0087 | 0.3661 |
| Trpv2 | TRPV2 | 6.56 | 7.16 | 1.51 | 0.0088 | 0.3666 |
| Trpc5 | TRPC5 | 10.29 | 9.92 | –1.29 | 0.0107 | 0.3858 |
| Kcnk1 | K_2P_1.1/TWIK-1 | 13.19 | 13.56 | 1.29 | 0.0167 | 0.4269 |
| Kcnj2 | K_ir_2.1/IRK1 | 5.8 | 6.4 | 1.52 | 0.0179 | 0.4312 |
| Kcnj14 | K_ir_2.4 | 5.2 | 4.82 | –1.3 | 0.0192 | 0.4338 |
| Kcnh7 | K_V_11.3/Erg3 | 10.4 | 9.77 | –1.55 | 0.0213 | 0.4406 |
| Kcnh2 | K_V_11.1/Erg1 | 7.95 | 8.2 | 1.19 | 0.0303 | 0.4811 |
| **Markers for reactive gliosis** | | | | | | |
| Vim | Vimentin | 10.35 | 11.12 | 1.82 | 3.36E-06 | 0.0125 |
| Gfap | GFAP | 13.87 | 14.85 | 1.97 | 0.0003 | 0.1744 |
| **Gene Symbol** | **Description** | **WT** | ***Hcn1^MI/+^*** | **Fold change** | ***P* value** | **FDR *P* value** |
| **Voltage-gated ion channels** | | | | | | |
| Kcnh3 | K_V_12.2/Elk2 | 8.57 | 8.12 | –1.36 | 0.0045 | 0.9722 |
| Kcnn4 | K_Ca_ 3.1/SK4 | 4.26 | 4.96 | 1.62 | 0.0073 | 0.9722 |
| Trpa1 | TRPA1 | 4.33 | 3.77 | –1.47 | 0.0108 | 0.9722 |
| Cacna1h | Ca_V_3.2 | 11.19 | 10.63 | –1.47 | 0.0161 | 0.9722 |
| Hcn3 | HCN3 | 5.28 | 4.53 | –1.67 | 0.0172 | 0.9722 |
| Kcnq3 | K_V_7.3 | 13.14 | 12.85 | –1.23 | 0.0225 | 0.9722 |
| Kcnk1 | K_2P_1.1/TWIK-1 | 13.19 | 13.56 | 1.29 | 0.0297 | 0.9722 |
| Kcnj4 | K_ir_2.3 | 8.28 | 7.73 | –1.46 | 0.0335 | 0.9722 |
| Trpc6 | TRPC6 | 7.78 | 8.94 | 2.24 | 0.0386 | 0.9722 |
| Kcna10 | K_V_1.10 | 4.61 | 5.03 | 1.33 | 0.0436 | 0.9722 |
| **Markers for reactive gliosis** | | | | | | |
| Vim | Vimentin | 10.35 | 10.62 | 1.21 | 0.0274 | 0.9722 |
| Gfap | GFAP | 13.87 | 14.14 | 1.21 | 0.1115 | 0.9722 |
